# An overview of Ghana’s mental health system: results from an assessment using the World Health Organization’s Assessment Instrument for Mental Health Systems (WHO-AIMS)

**DOI:** 10.1186/1752-4458-8-16

**Published:** 2014-05-04

**Authors:** Mark Roberts, Caroline Mogan, Joseph B Asare

**Affiliations:** 1 The Kintampo Project, Accra, Ghana; 2 Department of Psychiatry, University of Ghana, Accra, Ghana

**Keywords:** Mental health, Ghana, WHO-AIMS

## Abstract

**Background:**

This survey provides data on the Mental Health System in Ghana for the year 2011. It supplies essential planning information for the implementation of Ghana’s new Mental Health Act 846 of 2012, a renewal of the Ghana 5 year plan for mental health and it contributes to international knowledge base on mental health. It provides a baseline from which to measure future progress in Ghana and comparison data for use in other countries. In addition to reporting our findings we describe and analyse deficiencies and strengths of the Ghana mental health system.

**Methods:**

We used the World Health Organization’s Assessment Instrument for Mental Health Systems (WHO-AIMS) to collect, analyse, and report data on the mental health system and services for all districts of the ten regions of Ghana. Data was collected in 2012, based on the year 2011.

**Results:**

In 2011, Ghana was a lower middle income country with a population of approximately 25 million. A mental health policy, plan and legislation were in place. Mental health legislation was outdated and no longer in line with best practice standards. Services were significantly underfunded with only 1.4% of the health expenditure going to mental health, and spending very much skewed towards urban areas. There were 123 mental health outpatient facilities, 3 psychiatric hospitals, 7 community based psychiatric inpatient units, 4 community residential facilities and 1 day treatment centre, which is well below what would be expected for Ghana’s economic status. The majority of patients were treated in outpatient facilities and psychiatric hospitals and most of the inpatient beds were provided by the latter. There were an estimated 2.4 million people with mental health problems of which 67,780 (ie 2.8%) received treatment in 2011. The were 18 psychiatrists, 1,068 Registered Mental Nurses, 19 psychologists, 72 Community Mental Health Officers and 21 social workers working in mental health which is unbalanced with an unbalanced emphasis on nurses compared to what would be expected.

**Conclusions:**

The main strength of the mental health system was the presence of a long established service with staff working across the country in outpatients departments and hospitals. The main weakness was that government spending on mental health was very low and the bulk of services, albeit very sparse, were centred around the capital city leaving much of the rest of the country with almost no provision. Service provision was dominated by nurses with few other professions groups present.

## Background

This survey was conducted to meet pressing needs for high quality information for the Mental Health System in Ghana for;

• Renewal of the 2007–2011 Mental Health Programme of Work

• To operationalise a recently passed new Mental Health Act

• To evaluate a community-based mental health practitioner programme being implemented via ‘The Kintampo Project’

• Strengthen community mental health provision via all of the above

Expected outcomes were;

• Planning information to improve the mental health system

• A baseline from which to monitor change and progress in implementing reform policies

• Increased country awareness of the importance of good quality information

• Increased awareness of mental health through involvement of community services, users, families and other stakeholders in the process of data collection and analysis.

Over 100 countries have participated in the WHO AIMS project [1] It was first conducted in Ghana in 2005 [2] but that survey was limited and relied on much extrapolation. This is the first mental health system survey in Ghana that has covered the whole country.

Ghana is a West African tropical country sharing boundaries with Togo to the east, La Cote D’Ivoire to the west, Burkina Faso to the north and the Gulf of Guinea to the south. The country covers 238,533 square kilometres. The population in 2010 was 24,392,000 million of which, 51% were living in urban areas [3].

The economy is dominated by agriculture, which employs approximately 40% of the working population. Ghana is one of the leading exporters of cocoa in the world and is a significant exporter of commodities including gold and timber. A recent discovery of oil in the Gulf of Guinea could make Ghana an important oil producer and exporter in the next few years.

In 2010, 37.3% of the population was less than 15 years old, 6.7% was above age 60 and 4.1% was above age 64. The life expectancy was 57 years for males and 64 years for females [4]. The literacy rate was 67.3% [5].

English is the official language of Ghana and is universally used in schools in addition to nine other local languages. The most widely spoken local languages are Ga, Dagomba, Akan and Ewe [6]. 71.2 percent of the population are Christian, followed by Islam (17.6%), traditional religion (5.2%), no religion (5.3%) [2].

Based on World Bank criteria, Ghana is a lower middle income country [7]. The average per capita Gross National Income in 2011 was US$ 1,410. However, in 2007 nearly 30% of the population were still living below the poverty line. As the Mental Health Gap Action Programme [8] has demonstrated, most of the global burden of mental, neurological and substance use disorders occurs in countries with low-income and lower middle-incomes. These countries not only have the highest need to tackle this burden but also the fewest resources to do so. There is a tendency to prioritize initiatives that target infectious diseases and reproductive health, leaving mental health with minimal resources (Prince et al [9]). Ghana is no exception to this.

Health care delivery in Ghana is provided by both public and private sectors. The Ministry of Health exercises control over the whole system including policy formulation, monitoring and evaluation. Under the public health system, service delivery is undertaken largely by Ghana Health Service, The Teaching Hospitals and the Catholic Health Association of Ghana (CHAG). In addition, other quasi- and non-government institutions, religion-based and statutory bodies are involved in health service delivery. The total health care expenditure in 2011 was 7.8% of GDP and per capita expenditure was US$ 114.

Ghana’s mental health sector is funded primarily by the government and is supplemented to a small extent by internally generated funds and donations. It is estimated that of the 24.3 million people living in Ghana, 2.4 million suffer from mental illness [10]. Tradition and a general lack of facilities results in many patients looking for help from informal health services such as traditional and faith based practitioners who offer varying quality of service and level of efficacy [2].

A new Mental Health Act (846 of 2012) passed in 2012 is re-focussing the way mental health services are to be provided, moving from an institutional model to a community-based approach. The new Act is also designed to combat stigma and discrimination against mentally ill people which is as rife in Ghana as elsewhere.

Although the new Act, along with other community mental health initiatives such as The Kintampo Project [11] provide new vigour and interest in mental health there has been insufficient comprehensive, aggregate in-country data for planning purposes meaning that without an intervention like the WHO-AIMS, it has not been possible to make reliable estimates of information about Ghana’s mental health system for planning needs.

This paper now reports and discusses the findings of the WHO-AIMS survey in Ghana conducted in 2012 for the year 2011.

## Methods

The WHO-AIMS Version 2.2 [12] was used to collect, analyse and report data on the mental health system and services for all districts of the ten regions of Ghana. Data was collected in 2012, based on the year 2011. The data collection phase was May-June 2012.

The WHO-AIMS tool has been developed to assess key components of a mental health system, thereby providing essential information to strengthen mental health systems. The instrument was developed following the publication of the World Health Report 2001 [13] which focused on mental health and covers the 10 recommendations made in the report. It consists of 6 domains, 28 facets and 156 items. The 6 domains are interdependent and conceptually interlinked. The domains are:

• Domain 1: Policy and Legislative framework

• Domain 2: Mental health services

• Domain 3: Mental health in primary care

• Domain 4: Human Resources

• Domain 5: Public education and links with other sectors

• Domain 6: Monitoring and research

Thirteen short questionnaires seeking specific information were generated directly from the 156 items in the WHO-AIMS document, each targeting specific respondents. The item number, characteristic and salient content of the questions were maintained. Each questionnaire targeted one of the following respondents:

• Chief Psychiatrist

• Director/Nurse Manager/Principal Nursing Officer of each mental hospital

• Director/Nurse Manager/Principal Nursing Officer of each outpatient service

• Head of each general hospital with an inpatient psychiatric service

• Head of each private psychiatric service

• Head of each community residential service

• Chief Pharmacist

• Head of Finance at Ghana Health Service (GHS)/psychiatric hospitals

• Director of Family/Public Health at GHS

• Head of Nursing and Midwifery Council/Medical and Dental Council/Directors of Nursing/Medical schools

• Director of Policy, Planning, Monitoring and Evaluation at the Ministry of Health

• Officer in Charge of Ghana School Health Education Programmes

• Director of Health, Ghana Police Service

• Officer in Charge of Statistics, Ghana Prison Service

As an unusual extension to the WHO-AIMS, for this project we developed separate questionnaires for traditional and faith based practitioners (‘fetish priests’ and religious ‘pastors’) using similar criteria in the questionnaires aimed at staff from the outpatient services. One traditional practitioner and one faith based practitioner from each of Ghana’s 10 regions were identified at random, thus providing us with 20 completed questionnaires.

Data was collected by ten pairs of data collectors (one pair of from each region of Ghana) consisting of one Community Psychiatric Nurse and one Community Mental Health Officer. They were trained to assist in the data collection for their region. Interviews were undertaken with the aforementioned respondents. Data was processed via a standardised WHO-AIMS 2.2. Excel spread sheet.

A draft report was prepared and discussed with the in-country Focal Point (the WHO-AIMS requires there to be a well-connected, senior and politically neutral person leading the survey, this person being referred to as the ‘in-country Focal Point’), UK Project Co-ordinator and Chief Psychiatrist for comments. Where information was lacking the Delphi technique was used. Once the initial draft report was ready, findings and further analyses were disseminated to key stakeholders in Ghana for consultation, refinement and contextualisation.

Permission to conduct this survey was granted by the Ministry of Health, Ghana and the Chief Psychiatrist of Ghana. It was funded by UK Department for International Development (DFID) and the Ministry of Health, Ghana.

The survey, data collection and analysis was led and undertaken by the Kintampo Project. The Kintampo Project, which was formed in 2007, is a partnership between the College of Health, Kintampo (Ghana) and Southern Health NHS Foundation Trust in Hampshire (UK). The Kintampo Project is increasing the community mental health workforce in Ghana by training new health professionals, and ensuring sustainability by supporting graduates with professional development.

## Results

### Mental Health Policy and Plans

Policy is formulated by the Ministry of Health and implemented through the Ghana Health Service. In 2011, a mental health policy that had last been revised in 1996 was in place. It did not cover the integration of mental health into primary care, downsizing psychiatric hospitals or the protection of human rights of patients but it did include the following WHO-AIMS components:

• Organization of services and developing community mental health services

• Human resources

• Involvement of users and families

• Advocacy and promotion

• Equity of access to mental health services across different groups

• Financing

• Quality improvement

• A monitoring system

The policy also included a list of essential medicines which had last been revised in 2004. The medicines included antipsychotics, anxiolytics, antidepressants, mood stabilizers and antiepileptic drugs.

The last revision of the mental health plan had been in 2007 (“2007-2011 Mental Health Strategy”) and contained a budget, timeframe and specific goals. However, by 2011 a lack of funds had prevented many of the goals being reached.

Ghana did not have an emergency/disaster preparedness plan for mental health.

### Legislation and Human Rights protection

In 2011, the mental health system operated on an outdated mental health law that had last been revised in 1972 (The Mental Health Decree NRCD). Although the Mental Health Decree NRCD contained WHO-AIMS components such as; voluntary and involuntary treatment, law enforcement and other judicial system issues for people with mental illness, mechanisms to oversee involuntary admission and treatment practices and mechanisms to implement the provisions of mental health legislation, it focused mainly on institutional care of the mentally ill and was not in accordance with contemporary international human rights standards regarding mental health care.

In 2011, the Commission on Human Rights and Administrative Justice (CHRAJ) was functioning on a national level to assess the human rights and protection of mental health patients. CHRAJ had the authority to oversee regular inspections of mental health facilities but they did not have the authority to review involuntary admission/discharge procedures or complaints investigation processes, nor to impose sanctions. In 2011 all three mental health hospitals in the country had at least one external review of the human rights protection of their patients by CHRAJ but only one of these facilities had specific training on the subject.

### Financing of mental health services

#### ***Public funding for mental health***

Figures from the Ministry of Health for 2011 showed a ring-fenced mental health budget for the three psychiatric hospitals of 4,516,163 Ghana Cedis (GhC) (without personal emoluments). However, the de facto spending on mental health in 2011 was GhC 5,656,974 because the funding that was initially approved was lower than what was actually required.

Almost 100% of the ring fenced budget was spent on the three psychiatric hospitals. Beyond this budget, mental health spending in Ghana was difficult to ascertain due to the integration of mental health into primary care where the delineations of services provided were more difficult to estimate. Money released for the psychiatric hospitals covered overhead costs, including basic medical supplies and service maintenance.

In total, the spending on mental health from the government was a minimum of 1.4% of the total health budget. As noted, the actual government spending on mental health was higher than this however because the only budget that could be isolated was that spent on the mental hospital, the rest could not be calculated.

#### ***Other funding for mental health***

In addition to public funding, mental health in Ghana is also funded by international development partners and to a small degree, by internally generated funds. A very small amount is also contributed by NGOs which purchase some medicines when the hospitals run out of government allocation. The additional moneys are pooled into communal hospital funds and distributed across services within hospitals.

Mental health services do not usually generate revenue, since most patients are too poor to pay the fees and by government policy mental health care is supposed to be free. As a result, mental health services are subsidised by this system of pooled internally generated funds.

Some mental health care is purchased directly by patients and their families via private services and the traditional/faith-based practitioner systems. Some patients are faced with having to buy their own medicines when government supplies run short. It was not possible to calculate the amounts spent these ways.

### Mental Health Services

#### ***Organization of mental health services***

In 2011, Ghana did not have a governing national or regional mental health body and mental health services were not organized into catchment or service areas. The responsibility for national organization of mental health services was vested in the Chief Psychiatrist as the national head who also served to directly advise the Minister for Health on mental health. There was a focal person for mental health located in the Institutional Care Division of the GHS, who integrated mental health care in the GHS institutions. The Chief Psychiatrist also coordinated the planning and organization of mental health activities at the national level. At the regional and district levels the Regional and District Coordinators of Community Psychiatric Nursing served as the coordinators.

#### ***Outpatient Services***

There were 123 mental health outpatient services in the country and although 14% of all patients treated in mental health outpatient units were 17 years or younger, there were no services reserved exclusively for children and adolescents.

A total of 57,404 patients were treated in outpatient facilities in 2011 (this figure is for individual patients treated, it does not include follow up appointments). This equates to 237 patients per 100,000 general population overall. Fifty four per cent of these patients were female. It is estimated that the average number of contacts per patient to these services was 4.99.

Diagnoses in outpatient services were primarily of schizophrenia (25%), mood disorders (10%), neurotic and stress related disorders (8%), substance misuse (7%), disorders of adult personality and behaviour (1%) and “other” diagnoses such as epilepsy, organic disorders, mental retardation (39%). Ten per cent of patients did not have a diagnosis.

Eighty-eight per cent of outpatient services provided clinic-based follow-up and fifty nine per cent had mental health mobile teams to conduct outreach clinics, although this was not routine practice. In terms of available interventions, approximately twenty per cent of the patients in outpatient services received psychosocial interventions and forty per cent of the facilities had at least one psychotropic medicine of each therapeutic class (anti-psychotic, antidepressant, mood stabilizer, anxiolytic, and antiepileptic medicines) available on site or at a near-by pharmacy all year round.

#### ***Day treatment facilities***

Day treatment services are a mid-way provision for patients who need some degree of daytime support but who do not require full inpatient care.

In 2011, Ghana had only one day treatment facility, which was located in its Western Region. This was a private facility run by the Catholic Church providing patients with a structured daily programme which included pastoral care, psycho-education, psychomotor skills, occupational therapy and leisure activities such as games and crafts. It was staffed by one therapist, one psychiatric nurse and one hospital assistant. In 2011 the service treated 18 patients (0.07 per 100, 000 general population), three of which were successfully discharged. Of all patients treated in the day treatment service, 22% were females and none were 17 years or younger. On average, patients spent two hundred and thirty nine days attending the day treatment unit.

#### ***Community-based psychiatric inpatient units***

A community based psychiatric inpatient unit is a facility that provides inpatient care for the management of mental disorders within a community-based facility. These are usually units located within general hospitals and they provide care to patients with acute problems. The period of stay is usually short (weeks to months).

There were seven community-based psychiatric inpatient units in the country with a total of 120 beds. Two were private facilities with the remaining 5 being connected to regional hospitals. Forty-seven per cent of admissions to community-based psychiatric inpatient units were female. Although there were no beds reserved exclusively for children, three per cent of the patients treated in community-based psychiatric inpatient units were 17 years or younger.

The diagnoses of admissions were primarily schizophrenia (21%), substance misuse (9%) and mood disorders (6%). 58% of patients did not have a diagnosis.

It was estimated that on average, patients spent 16 days in hospital per admission. Records of physical restraint and seclusion were available in four out of the seven facilities. These indicated that approximately 10% of patients were physically restrained and/or secluded.

Fifty seven per cent of community-based psychiatric inpatient units had at least one psychotropic medicine of each therapeutic class (anti-psychotic, antidepressant, mood stabilizer, anxiolytic, and antiepileptic medicines) available in the facility and it was estimated that between 21-50% of patients received psychosocial interventions whilst admitted.

#### ***Community Residential Facilities***

In 2011, Ghana had four long stay (“residential”) units that were based away from psychiatric inpatient units and psychiatric hospitals. In total, these provided 112 beds and treated 122 patients with 46% of patients being female. The average number of days patients spent in these services was 365. Although none of the beds were reserved solely for children, 2% of patients treated in community residential facilities were 17 years or younger.

#### ***Psychiatric Hospitals***

There were three psychiatric hospitals in the country with a total of 1322 beds. Two were located in the capital city and the other located on the coast in the Central Region. All the hospitals had mental health outpatient facilities.

Only one of the hospitals had a children’s ward and this contained 15 beds. However, children were also accommodated in the other two hospitals if there was a need. In 2011, 1% of patients treated in the psychiatric hospitals was 17 years or younger.

The number of admissions to psychiatric hospitals in 2011 was 7993 with 32% being female. The diagnoses of admissions to psychiatric hospitals were primarily schizophrenia (32%), substance misuse (26%) and mood disorders (19%). A further 1% of patients had a diagnosis of neurotic, stress related disorder and 6% were classed as having an “other” diagnoses such as epilepsy or organic mental disorders. No patients were diagnosed with personality disorder.

The number of beds in psychiatric hospitals decreased by 13% between 2006–2011. Accurate data regarding the length of stay of patients in psychiatric hospitals was difficult to estimate due to insufficient record keeping. However, based on data from 2 of the 3 hospitals, 77% of patients spent less than one year in hospital, 11% spent between 1–4 years, 5% spent between 5–10 years, and 7% spent more than 10 years in psychiatric hospitals. These figures are extrapolated from a sample 4397 of patients.

Records for the cumulative number of days patients spent in hospitals was unavailable due to the aforementioned reasons. Data from one hospital (2753 patients) showed an average number of days spent in psychiatric hospitals to be 23.6.

In terms of treatment it was found that approximately 19% of patients in psychiatric hospitals received one or more psychosocial interventions.

One hundred per cent of psychiatric hospitals had at least one psychotropic medicine of each therapeutic class (anti-psychotic, antidepressant, mood stabilizer, anxiolytic, and antiepileptic medicines) available in the facility all year long. However, it was often the case that newer medicines (such as Olanzapine) would deplete throughout the year meaning that patients would have to continue on medications that they were not initially prescribed.

None of the psychiatric hospitals kept records on the number of patients who were restrained or secluded. However, estimated figures showed that over 20% of patients were restrained or secluded in 2011.

### Forensic and other residential services

There were 79 dedicated inpatient beds for forensic patients across the three psychiatric hospitals, representing approximately 6% of the total beds. In 2011 there were 148 forensic admissions to psychiatric hospitals (less than 2% of the total).

Other residential services included 10 services across the 10 regions for children under 17 with intellectual disabilities and one private school for children with intellectual disabilities located in the capital city. There were two private residential services for people with substance abuse and one private hospital that had a detoxification unit. All were located in the capital.

One hospital had beds dedicated to old age patients (18 in total). There were no other specialised mental health services for older people, or people with conditions such as dementia.

### Distribution of inpatient beds

The density of psychiatric beds in and around the capital city was 7.23 times the density of beds in the rest of the country. The solely coastal and city based located of the hospitals prevented easy access for rural populations and those living away from the coast.

### Mental health in primary health care

There were both physician based primary health care (PHC) and non-physician based PHC clinics available and it was estimated that around 1-20% had access to assessment and treatment protocols for key mental health conditions. These were offered in the form of “Standard Treatment Guidelines” that were available in booklet form.

Primary care nurses and non-doctor/non-nurse primary health care workers, by policy were not allowed to prescribe psychotropic medications under any circumstances. However, primary health care doctors were allowed to prescribe psychotropic medications without restrictions as were Medical Assistants and Community Psychiatric Nurses.

With regard to the availability of psychotropic medications, 81-100% of the physician based PHC clinics and 1-20% of the non-physician based PHC clinics had at least one psychotropic medicine of each therapeutic category (anti-depressant, anti-psychotic, mood stabilizer, anxiolytic and anti-epileptic) available all year round.

There were no formal avenues for professional interaction between primary health care staff and other care providers so it is unknown how many primary health care doctors had interactions or made referrals to mental health professionals in 2011. However, it was estimated that between 21-50% of non-physician based PHC providers made referrals to higher levels of care at least once per month.

In 2011, three per cent of undergraduate training for medical doctors was devoted to mental health compared to ten per cent for State Registered Nurses (SRNs). Ghana Health Service reported that no primary health care staff received any refresher training in mental health in 2011.

### Informal primary health care (Faith-based and traditional practitioners)

#### ***Faith-based facilities***

In Ghana some religious leaders operate ‘prayer camps’ and conduct ‘healing’ in various places including the prayer camps. For this survey we refer to these practitioners as ‘faith-based’ practitioners.

Across the 10 faith-based facilities surveyed, 1253 people were ‘treated’ in 2011, 37.5% of which were female and 8% children or adolescents. The average number of contacts was 1.18 per person.

Although ICD 10 was not used by faith-based practitioners, each stated that most of the people they ‘treated’ had diagnoses of some kind. These included schizophrenia (25%), substance or alcohol misuse (19%), mood disorders (6%), epilepsy (5%) and “Other mental health disorders” (45%). Some of the diagnoses in the “other” category included “witchcraft” (0.6%) and “spiritual attack” (0.7%).

In terms of available interventions, alongside spiritual practices, 56% of faith based healers administered medications and 22% offered herbal remedies. Out of all of the people treated, 57.5% were restrained at least once across 8 of the facilities. The remaining two stated they did not use restraint or seclusion.

One of the ten practitioners had received training in psychiatric care in 2011. This had been provided by the NGO “Basic Needs”. Two of the facilities made referrals to psychiatric services in 2011.

#### ***Traditional practitioners***

In Ghana traditional healers known as ‘fetish priests’ offer treatments and for this survey we refer to these practitioners as ‘traditional’ practitioners.

Across the 10 traditional practitioner facilities sampled, 749 people were ‘treated’ in 2011. 39% of these were female and 8% were children or adolescents. The average number of contacts per person was 1.19.

Akin to the faith based facilities, ICD 10 diagnoses were not used but most people treated by traditional practitioners had been given diagnoses of some kind. These diagnoses included substance or alcohol misuse (19%), schizophrenia (15%), mood disorders (10%), epilepsy (8%) and “other mental health disorders” (48%).

In terms of available interventions, alongside using herbal remedies, 40% used “rituals” and 30% used spiritual practices. Out of all of the people treated, 41.1% were restrained at least once in 9 of the facilities.

Like in the faith based facilities, one of the traditional healers had received training in psychiatric care in 2011.

### Human Resources

The total number of staff working in mental health facilities was 1887. The breakdown by profession was: 18 psychiatrists (0.07 per 100,000 population), 31 other medical doctors not specialized in psychiatry (0.13 per 100,000), 1,256 nurses (5.19 per 100,000), 19 psychologists (0.08 per 100,000), 21 social workers (0.09 per 100,000), 4 Occupational Therapists (0.02 per 100,000) and 546 other mental health workers (2.25 per 100,000) which included auxiliary staff, health assistants, medical assistants, professional and paraprofessional psychosocial counsellors.

Most psychiatrists worked in more than one type of facility. There were 9 psychiatrists in outpatient services, nine in community-based psychiatric inpatient units and 8 in psychiatric hospitals. As for other medical doctors not specialized in mental health, 20 worked in outpatient facilities, 5 worked in community-based psychiatric inpatient units and 5 worked in mental health hospitals. As for nurses, 347 (31 SRN, 287 RMN and 29 EN) worked in outpatient services, 57 (11 SRN, 33 RMN and 13 EN) worked in community-based psychiatric inpatient units and 848 (5SRN, 748 RMN and 95 EN) worked in mental health hospitals.

Twenty-seven psychosocial staff (psychologists, social workers and occupational therapy assistants) worked in outpatient services, 9 in community-based psychiatric inpatient units and 11 in mental health hospitals. For other health or mental health workers, 122 worked in outpatient services (including 13 qualified.

Community Mental Health Officers, 1 Psychotherapist, 1 Non specified therapist and 107 Medical Assistants, health extension workers or health care assistants), 26 worked in community-based psychiatric inpatient units and 366 worked in mental health hospitals.

The number of health professionals who graduated in 2011 in academic and educational institutions was 2494 (10.30 per 100,000 population). These included 1 Psychiatrist (0.004 per 100,000), 283 other medical Doctors not specialized in psychiatry (1.17 per 100,000), 1,871 State Registered Nurses (SRNs) (7.72 per 100,000), 334 Registered Mental Health Nurses (RMNs) (1.38 per 100,000), 5 Clinical Psychologists (0.02 per 100,000). No Social Workers with at least 1 year training in mental health care or occupational therapists graduated in 2011.

In 2011 a new generation of community mental health workers unique to Ghana were being trained and introduced to help shift the focus away from large psychiatric hospitals to the community where it is most needed. Two new training programmes had been developed – Degree in Community Medicine and Clinical Psychiatry producing Clinical Psychiatric Officers (CPO) and Diploma Community Mental Health producing Community Mental Health Officers (CMHO). The two year CPO course was producing mental health practitioners who can practice independently where there are no psychiatrists, or complement psychiatrists where there are and the one year CMHO course was producing frontline community level mental health workers to assist Community Psychiatric Nurses.

In late 2011, 72 CMHOs graduated and started working around the country and the first batch of CPOs graduated in 2012.

### Consumer associations, family associations and NGO’s

In 2011 there were eight consumer associations/NGOs in Ghana. These included Mental Health Society of Ghana (MEHSOG), The Ghana Mental Health Association, Mindfreedom, Alcoholics Anonymous, The Epilepsy Association, BasicNeeds, World Vision and Psycho-Mental Health International. Data about the numbers of members in these associations could not be obtained.

BasicNeeds, MEHSOG and Mindfreedom had all been involved in the formulation or implementation of mental health policies, plans, or legislation in Ghana. Basic Needs and World Vision were also involved in individual assistance activities such as counselling, housing, or support groups.

### Public education and links with other sectors

In 2011, Ghana did not have an overall coordinating body that supervised public education and awareness campaigns on mental health. However, this area of activity was overseen by a number of different organizations including the Ghana Mental Health Association.

Since 2006, Government agencies, NGOs, professional associations, and international agencies had all promoted public education and awareness campaigns. These campaigns targeted the general population and women. In addition, there were public education and awareness campaigns targeting professional groups including health care providers and those working in the complimentary/alternative/traditional sector.

### Links with other sectors

In terms of support for child and adolescent mental health, none of the primary and secondary (high) schools had either a part-time or full-time mental health professional but a few (1-20%) primary and secondary schools had school-based activities to promote mental health and prevent mental disorders. These were usually offered through teaching sessions by Community Psychiatric Nurses.

Regarding mental health activities in the criminal justice system, a survey by the Officer in charge of statistics at Ghana Prison Service showed that the percentage of prisoners with psychosis was 0.7%. The corresponding percentage for mental retardation was less than 2%. It was estimated that between 1–20% of prisons had at least one prisoner per month in treatment contact with a mental health professional. It was usual practice that if a prisoner was to become mentally unstable whilst imprisoned, they would be sent to one of the psychiatric hospitals until they were fit to return.

No police officers and no judges or lawyers had participated in any kind of training on mental health in the preceding 5 years.

In terms of financial support for patients, no mental health services had access to programs outside the mental health facility that provide outside employment for patients with severe mental disorders. However, one community residential facility had onsite training and workshops for patients to learn certain trades whilst residing at the unit. Items produced were sold to visitors.

### Mental Health Research

A PubMed search for the years 2006–2011, showed that one per cent of all health publications from the country were on mental health. The mental health research focused mainly on policy, programmes and financing/economics (38%). Other papers included service research (29%), epidemiological studies in clinical samples (19%), epidemiological studies in community samples (9%) and psychosocial interventions (5%).

## Discussion

### Weaknesses of the mental health system

The main weakness was that government spending on mental health was very low. The bulk of the services were centred on the heavily populated capital city of Accra leaving much of the rest of country with only very sparse provision. Development of the mental health system had been neglected despite pressure and campaigning from able mental health leaders in the country and the mental health law passed in 1972 had never actually been implemented. Service provision was dominated by nurses with few other professional groups present in any number. When compared to other LIC and LMIC countries Ghana was at the LIC level although it had become officially LMIC in 2011.

#### ***Weaknesses in Policy and Legislative Framework***

• Insufficient funding had compromised effective service delivery particularly area coverage.

• There was a lack of regional and district management structures for mental health with multiple negative consequences including very inadequate systems for planning, monitoring, service and quality improvement.

• The system was too strongly focused on inpatient care.

• There was very little use of legislation to regulate detention of patients thus widespread breaching of human rights.

• The supply of psychotropic medications was not consistent or uniform in coverage.

• There was a lack of policy and regulation concerning the practice of psychiatry by traditional and faith based practitioners.

• There was insufficient use of clinical guidelines even where they exist.

• There was inadequate legal and financial support for people with mental disorders in the areas of employment and housing.

• There was only low potential for Internally Generated Finds (IGF) as service users were usually poor.

#### ***Weaknesses in Mental Health Services***

• Insufficient in-patient facilities in the Regions and Districts was putting burden on families who had to travel long distances in search of treatment.

• There was overcrowding in some of the inpatient facilities.

• There was a very low level of community based rehabilitation facilities.

• Management of substance abuse was deficient outside psychiatric institutions.

• There was inequitable distribution of resources such that nearly all the resource was provided via 3 hospitals located in large urban centres in the south.

• The few rehabilitation units which exist were ‘blocked’ by long stay patients.

• There were high rates of restraining (mechanical and/or non-mechanical) and secluding disturbed patients.

• Patients were being secluded and restrained when not formally detained.

• Supply of community mental health facilities (eg office and clinic space) and resources (eg medication supplies and transport) to support community mental health practice was very insufficient.

• There was a lack of services specifically for children.

• There was only one day treatment centre in the country whereas there should have been several hundred.

• The number of community based psychiatric inpatient units was very low.

• The number of community residential facilities was very low.

• There were insufficient specialist services, particularly for children, the elderly, learning disabled, forensic and substance misusers.

#### ***Weaknesses in Mental Health in Primary Health Care***

• Mental health services had to be provided by inadequately trained staff, such as generic health workers.

• There was a lack of referral systems for healthcare workers to know how to refer cases into the mental health system.

• Traditional practitioners were restraining patients without legal authority to do so, which was breaching human rights.

#### ***Weaknesses in Human Resources***

• There was a heavy over reliance on nurses with very few other types of specialist.

• There was insufficient manpower particularly, psychiatrists, psychologists, occupational therapists, workers trained for community mental health practice and psychiatric social workers.

• There were insufficient incentives for staff working for mental health.

• Mental health staff and primary health care workers were hardly doing any refresher training at all.

• There was hardly any training of mental health workers on human rights.

• The balance of treatment for patients was too strongly focussed on medication rather than psychosocial interventions and prevention.

• The amount of postgraduate training taking place in psychiatry for doctors was very low.

• Very few doctors choose to specialize in mental health.

#### ***Weaknesses in Public education and links with other sectors***

• There was insufficient public education which was likely to adversely affect acceptance of the mentally ill in the community and their rehabilitation.

• There was no coordination of public education/awareness raising campaigns etc. for mental health.

• Criminal justice personnel had had no mental health training.

• There was inadequate information on the prevalence of mental health problems in prisons.

#### ***Weaknesses in Monitoring and Research***

• The mental health information system was not adequate, it was not being used consistently enough and data was not being aggregated and reported.

### Strengths of the mental health system

Despite significant weaknesses, Ghana’s mental health system had strengths. The main strength was the presence of a long established service with staff working across the country in outpatient departments and hospitals. The service was being led by an able chief psychiatrist supported by other senior leaders. There were 3 large fully active mental health hospitals and a new mental health act had been passed with the power to refocus mental health services into the community and bring in robust structures for protection of human rights. Specifically, the provisions of the new Act included:

• Improving access to in-patient and out-patient mental health care in the communities in which people live.

• Human rights protection through regulation of mental health practitioners in both the public and private sectors and traditional healers too, everywhere in communities and hospitals.

• Combating of discrimination and stigmatization against people with mental illness and promoting their human rights.

• Promoting voluntary treatment.

• Clearly defining and limiting the circumstances under which treatment may be given to people with mental disorders without their consent.

#### ***Strengths in Policy and Legislative Framework***

• There were documented Mental Health Programmes with activities which were championed by the Chief Psychiatrist.

• Mental Health service planning was taking place and 5 year mental health plans were being produced.

• A budget line for mental health existed at the Ministry of Health even though it was not sufficient.

• Government was providing free treatment and accommodation for the mentally ill.

• Psychotropic medication was available.

• The country had passed a new modern Mental Health Act in 2012.

• Hospitals had been inspected for Human Rights monitoring and some staff had had training

• Standard treatment guidelines were available

• A national formulary which includes psychoactive medication was available.

• Government recognised the mental health needs of the population and was supporting mental health service improvement.

#### ***Strengths in Mental Health Services***

• Mental health was to some extent decentralized

• There were some facilities for helping people with mental health problems in outpatients, inpatients and in the community. There were also traditional treatments for the mentally ill which were safe for some disorders.

• Structures for providing mental health treatment and aftercare in the community were available.

• A five-tier decentralised health system existed which mental health can integrate with.

#### ***Strengths in Mental Health in Primary Health Care***

• Primary care practitioners were providing mental health services.

• Working relationships existed with faith based and traditional healers.

#### ***Strengths in Human Resources***

• There were some psychiatrists, although very few.

• A range of practitioners existed including psychologists.

• Institutions for training doctors and nurses were available even to postgraduate level.

• Training programmes were in place producing middle level specialists in mental health.

• Opportunities existed in Ghana for postgraduate specialisation in mental health.

#### ***Strengths in Public education and links with other sectors***

• Links existed with overseas mental health specialists and services, particularly in the UK and US.

• NGOs for mental health and service user organisations existed.

#### ***Strengths in Monitoring and Research***

• Mental health service informatics was good enough to be able to produce data for assessing the system.

• Research on mental health was taking place.

## Conclusions

Ghana’s mental health system is now in the control of a new Mental Health Authority established in November 2013 and they are responsible for implementing the Mental Health Act passed in 2012.

From the data collected in this survey, we have identified some top level priorities that would be most likely to assist the realisation of the detailed recommendations. Firstly, the Mental Health Board need to develop the Legislative Instrument for the Act, for Parliament and also the Board needs Parliament to agree a budget for implementation of the Mental Health Act. Secondly, mental health structures described in the Act need to be put in place as soon as possible at all levels across districts, regions and nationally. The Act also needs to be introduced to the public and stakeholders need to be educated on its application. Finally, the commissioning of an expert team to work in collaboration with MoH and health providers to produce evidence based mental health improvement plans with short, medium and long term goals should be considered. These plans should take into account needs in relation to the implementation of the Mental Health Act and needs from the findings of the WHO- AIMS survey in relation to Ghana’s status as a lower-middle income country. The work of the proposed expert team with the implementation of a staged project plan which is well managed and supported by experts in project management should also be considered.

Additional recommended next steps that should be taken into account when producing the mental health improvement and action plans are also detailed below. These have been based upon the six domains of the WHO AIMS tool.

### Policy and Legislative Framework

In 2011, there was insufficient funding for mental health services in Ghana. Therefore, the budget for these services needs to be increased through dedicated funding which should be properly managed for the expansion of mental health services in the community. Furthermore, in the spirit of decentralization, measures need to be put in place to track funds released to the service to ensure work is integrated and monitored.

The lack of legislation used to regulate the detention of patients in 2011 means that it is essential that the provisions in the Mental Health Act 846 of 2012 should be specifically implemented as soon as possible in order to enhance human rights. In addition, the organizational structure of the Mental Health Authority should include a division for Quality Assurance and a division for Monitoring and Evaluation. Plans for training/awareness creation and monitoring should also be produced for the changes that will occur from the implementation of the Mental Health Act.

Due to the highlighted inconsistencies in the supply of psychotropic medications, during the initial stages of the implementation of the Act, at least for the next 5 years, selected psychotropic medication should be included in the National Health Insurance Scheme and subsidy should be given to the new generation psychotropic drugs by the Authority.

Finally, the Mental Health Authority should establish a system to maintain oversight of and to coordinate all the different groups working alongside government contracted and private mental health service providers in the country. These ‘groups’ include consumer associations, family associations, NGO’s and groups and individuals from overseas. The task should include the coordination of the efforts of these ‘groups’ and ensuring their work is in line with the mental health strategy of the country. There should be some visibility of the different groups and what they do perhaps via a website or an easily available and regularly updated ‘register’.

### Mental Health Services

In 2011, there was inequitable distribution of resources around the country and nearly all mental health care was provided by the three psychiatric hospitals in the south of the country. This discrepancy should be addressed in the implementation of the Act through decentralization and refocusing on community care. The paradigm shift from institutional to community care as envisaged by the Mental Health Act, calls for downsizing or total abolition of the Accra Psychiatric Hospital and the retraining of the staff for community based activities. The policy of de-emphasizing institutional care and the provision of mental health services in the regions and districts should also be continued. This policy calls for existing staff to retrain for community mental health and other areas.

In 2011, there was only one day treatment facility in the country. Because these services help to provide care- givers with some reprieve and also prevent inpatient care to some extent, as well as offering accessible opportunities for rehabilitation and improvement on social and communication skills, it is recommended that there should be at least one day treatment facility in each region. Many more than this will eventually be needed but at least one per region would be in the right direction.

In 2011, forensic wards in the psychiatric hospitals were inadequate and were staffed by nurses who have no adequate training for the job, which presents unacceptable risks to patients, staff and visitors. This should be rectified by providing proper facilities and training for all staff managing such patients and the Mental Health Authority should pursue the provision of a forensic facility in a maximum security prison.

Finally, mental health services for children and adolescents in 2011 were almost non-existent. This group are the future adult citizens of the country and they represent almost 40% of the population. They accordingly require a concentrated focus for mental disorder prevention programmes as well as treatment for those who are already afflicted by mental health problems. There should be at least one comprehensive adolescent mental health service in each region providing, in-patient, outpatient and rehabilitation facilities where counselling, social skills training and prevention programmes can be administered. Child and adolescent mental health should be considered as the first area to develop as a specialism once all practitioners have developed the requisite basic skills in it. Specialized treatment facilities and staffing should also be made available for the aged.

### Mental Health in Primary Health Care

In order to successfully decentralize and integrate mental health services, programmes should be put in place to train new non-mental health workers in the Primary Health Care system and existing primary care practitioners should be provided with specific training in mental health for public education, case detection, support and referral of cases. Treatment protocols and algorithms for Primary Health Care providers should be developed and these should include recommended appropriate psychotropic medication for use at the Primary Health Care level. The medication in these protocols should be available at all times. In addition, the essential drug list needs to be updated and a policy developed to guide the availability of psychotropic drugs at various levels commensurate with levels of training of practitioners.

Because of the lack of communication between primary care and mental health, referral systems should be formalised with standard national procedures so that everyone knows the system and what to do. This should become possible with the establishment (via the Act) of Regional and District Mental Health Sub-Committees, although the Board will still need to lead this for it to become established.

In 2011, many people in the country still used non-orthodox mental health practitioners. Because of this a series of meetings should be organized with traditional and faith based healers to harmonize their integration and their practices in line with the Mental Health Act. Clear policy guidelines for the practice of non-orthodox mental health practitioners should also be produced and traditional and faith based healers should be trained on the new Mental Health Act, particularly their obligations in relation to the human rights of patients.

### Human Resources

In 2011, there was a heavy over-reliance on nurses in mental health. Therefore, the human resource base should be expanded at all levels particularly, Psychiatrists, Clinical Psychologists, Psychiatric Social Workers and Occupational Therapists.

Special incentives should be considered for mental health practitioners to improve the manpower situation. This should include incentives to attract personnel into the training facilities in the country and to recruit others from the Diaspora. This has been tried and tested to good effect in some other countries, for example in the UK where very attractive pension allowances were introduced to attract doctors to become psychiatrists and even to this day mental health staff receive a ‘risk allowance’ for working in the specialty despite the fact that working in mental health in the UK is vastly safer than it is in Ghana.

The vision and now, practice, of training middle level personnel to help cover the Regions, Districts and Sub-Districts, should be vigorously supported. The College of Health at Kintampo should be adequately resourced for mental health training and priority should be given to training more Community Mental Health Officers and Clinical Psychiatric Officers.

### Public education and links with other sectors

In 2011, there was no legislation to protect the mentally ill with regards to employment, accommodation and access to treatment which should include access to physical health care treatment for those who cannot afford to be part of the National Health Insurance Scheme. The Mental Health Authority should ensure there is implementation of legislation and should also consider the creation of centres that will employ the mentally ill, stimulating self-sufficient employment e.g. Industrial Rehabilitation Centres. In addition, advocacy groups apart from the Mental Health Act statutory Visiting Committees should be encouraged to set up for the benefit of the mentally ill.

Because of insufficient public education about mental illness in the country, a Division or Unit should be created at the Mental Health Authority to coordinate continuous mental health promotion. Targeted efforts to reduce stigma should be pursued vigorously.

### Monitoring and research

In 2011, record keeping and data collection was inadequate and inconsistent. Therefore, health information systems should be improved to facilitate data collection and analysis. Information systems should be properly managed and there should be training in recording and keeping of records.

## Competing interests

The authors declare that they have no competing interests.

## Authors’ contributions

MR was the Principal Investigator for this project. He conceived the idea of this work & is the corresponding author. CM (Lead researcher) collected and analyzed data, with input from other colleagues working on the project. Together with MR, both CM and JB (in country focal point) participated in the design of the study and all have contributed to the alignment of this paper. All authors read and approved the final manuscript.
